# A systematic review and meta-analysis of the comparison of laparoscopic radiofrequency ablation to percutaneous radiofrequency ablation for hepatocellular carcinoma

**DOI:** 10.3389/fonc.2025.1559343

**Published:** 2025-03-11

**Authors:** Ya-Qiong Wang, Zhen-Kun Tan, Zha Peng, Hai Huang

**Affiliations:** ^1^ Hepatobiliary Surgical Department, The Second Affiliated Hospital of Guangxi Medical University, Nanning, Guangxi, China; ^2^ Graduate Institute, Guangxi Medical University, Nanning, Guangxi, China; ^3^ Hepatobiliary Surgical Department, Wuming Hospital of Guangxi Medical University, Nanning, Guangxi, China

**Keywords:** meta-analysis, hepatocellular carcinoma (HCC), laparoscopic radiofrequency ablation, percutaneous radiofrequency ablation (PRFA), radiofrequency ablation (RFA)

## Abstract

**Background:**

The comparative evaluation of laparoscopic and percutaneous techniques for liver radiofrequency ablation remains unexplored. This study aims to determine the most effective ablation technique and patient selection for hepatocellular carcinoma (HCC) by analyzing the efficacy and safety of laparoscopic radiofrequency ablation (LRFA) versus percutaneous radiofrequency ablation (PRFA).

**Methods:**

Two investigators (Y-QW and PZ) independently performed a literature search in the Cochrane Library, PubMed, Web of Science and Embase databases. Study quality was assessed using the Newcastle–Ottawa Scale or Cochrane risk-of-bias tool. Meta-analysis was conducted with Review Manager 5.4, applying either fixed- or random-effects models depending on study heterogeneity. The chi-square test (χ²) and I² statistics were employed for heterogeneity analysis.

**Results:**

Eight publications involving 1059 patients were included. Among them, 456 underwent LRFA and 603 underwent PRFA. LRFA showed a significantly better 3-year RFS than PRFA (OR: 1.89, 95% CI: 1.27-2.83, p = 0.002), the incidence rate of local recurrence was significantly fewer in the LRFA group (OR: 0.40, 95% CI: 0.23-0.69, p = 0.0010), but the postoperative hospital stay time was slightly shorter in the PFRA group (MD = 1.30; 95% CI 0.26 to 2.35; p=0. 01). Patients in the LRFA group had no significant difference in total postoperative complications, ablation success rates, overall survival (OS) and 1,5-year disease-free survival (DFS).

**Conclusion:**

Both LRFA and PRFA are effective treatments for HCC. LRFA shows better oncologic outcomes, including lower local recurrence and improved mid-term DFS. PRFA is simpler, less invasive and shorter hospital stays. The choice should be tailored to individual patient needs, considering tumor characteristics, comorbidities, and available expertise. Future research should focus on large-scale, prospective trials to validate these findings.

**Systematic review and registration:**

https://www.crd.york.ac.uk/PROSPERO/, identifier CRD42024601797.

## Introduction

Hepatocellular carcinoma (HCC) is a primary liver malignancy, accounting for more than 90% of all liver tumors (1). In China, HCC exhibits high incidence and mortality rates, ranking among the top malignant tumors nationwide (2). Globally, the incidence of HCC is increasing annually, with a rising trend observed in recent years (3). In terms of incidence and mortality rates, HCC ranks 6th and 4th, respectively, among all malignant tumors (4). According to estimates, approximately 840,000 new cases and 780,000 deaths occur annually (5).

Hepatocellular carcinoma has potential curative treatments in the form of liver transplantation, liver resection (LR) and local ablation therapies (6). Liver transplantation is considered the most effective treatment option for patients who fulfill the Milan criteria. However, the widespread use of this technique is significantly hampered by the critical shortage of donor organs (7). LR remains the first-line curative treatment for many HCC patients. However, only 5-15% of patients are suitable candidates for resection due to various contraindications such as multifocal tumors, unresectable locations, and insufficient hepatic reserve (8).

In recent years, radiofrequency ablation (RFA) has gained widespread acceptance as a local thermal ablation method owing to its technical feasibility, safety, effective local tumor management, and minimally invasive characteristics (9). RFA can be performed via percutaneous or laparoscopic approaches. When considering treatment options for HCC, the debate between LRFA and PRFA continues. A multitude of studies have been undertaken, but some have been unable to definitively demonstrate the superiority of one treatment option (10, 11). Meta-analysis combines the results of multiple studies to provide a more comprehensive understanding of a particular research question (12). It is necessary to evaluate the efficacy and safety of RFA from the perspectives of percutaneous and laparoscopic approaches.

## Methods

This systematic review followed the guidelines for the “Preferred Reporting Items for Systematic Reviews and Meta-Analyses” (PRISMA) (13). We registered our protocol with PROSPERO (ID: CRD42024601797).

### Inclusion and exclusion criteria for studies

#### Participants

We included patients with HCC meeting the Milan criteria (a single tumor ≤5cm or up to 3 nodules ≤3cm) and preserved liver function. The inclusion criterion for tumor size was ≤5cm due to the poor efficacy of RFA in treating HCC larger than 5.0 cm, regardless of whether single-electrode conventional RFA or multi-electrode RFA was applied (14). We excluded patients with extrahepatic metastases, vascular invasion, or a history of previous or concurrent malignancy.

#### Interventions

We compared LRFA with PRFA and LRFA as an intervention. LRFA is a surgical procedure that involves several steps. First, patients are administered general anesthesia, and tumor locations are determined through preoperative imaging studies such as CT or MRI. During the surgery, a laparoscope is inserted into the abdominal cavity, and a pneumoperitoneum is established, typically using CO_2_ gas at a pressure of around 1.3 kPa. Intraoperative laparoscopic ultrasound (LOUS) is then used to locate the tumor, and any adhesions are separated to protect surrounding organs. An appropriate puncture site is selected, and a cooled radiofrequency needle is used to perform the ablation, with treatment time and power adjusted based on the tumor size and location. The temperature of the tumor is maintained above 60°C, and each treatment session lasts between 10 to 15 minutes. For tumors smaller than 3 cm, a single-point, single-session puncture is used, while for larger tumors, multiple points and overlapping punctures are employed. Intraoperative assessment is conducted using LOUS to ensure that the ablation zone adequately covers the tumor and an additional 0.5 to 1.0 cm of surrounding liver tissue. After the procedure, the needle track is heated and coagulated to prevent bleeding and track seeding. Postoperative assessment includes blood tests on the first day after surgery and a contrast-enhanced CT scan one month later to evaluate the ablation effect. Regular follow-ups are conducted to monitor patient survival and recurrence rates.

#### Comparison

PRFA also involves a series of steps. Patients receive general or locate anesthesia, and tumor locations are confirmed through imaging studies. The procedure begins with the use of an external liver probe to determine the tumor’s position, often utilizing fusion imaging techniques that combine ultrasound with CT or MRI for precise localization. A puncture needle is inserted using a puncture frame, ensuring that the puncture site and angle are carefully selected to avoid direct tumor puncture. Similar to LRFA, a radiofrequency needle is used, and the tumor temperature is maintained above 60°C during treatment sessions. The approach for tumors smaller than 3 cm is the same as in LRFA, while larger tumors require multiple points and overlapping punctures. Intraoperative assessment is performed using ultrasound to confirm the ablation effect, ensuring that the zone covers the tumor and surrounding liver tissue adequately. The needle track is also managed to prevent complications. Postoperative assessments include blood tests and a contrast-enhanced CT scan one month after the procedure, followed by regular follow-ups to monitor patient outcomes.

#### Outcome

The primary outcome we focused on were OS and DFS. Secondary outcomes were ablation success rates, Local recurrence rate, postoperative hospital stay time, and overall complications. OS was defined as the length of time from the start of ablation to the date of the death or the last follow-up. DFS was defined as the length of time that the patient survived without any signs of HCC after ablation. Ablation success was defined as the complete eradication of the tumor, confirmed by high-quality cross-sectional contrast-enhanced imaging. Local recurrence was defined as any new lesion within or adjacent to the ablated area. Postoperative complications were evaluated according to the Clavien-Dindo classification. Hospital stay was defined as duration of patient admission for treatment and recovery.

#### Studies

For our analysis, we considered randomized controlled trials (RCTs) and non-randomized trials (NRTs) that have been published in any language.

### Exclusion criteria

#### Irrelevant literature

Lack of the required data in the Studies or statistical methods that are unreasonable.

Reviews, studies in animals, Case reports, letters, conference abstracts, and laboratory studies

### Literature search

We conducted a systematic search of the PubMed, Embase, Web of Science, and Cochrane Library databases to identify RCTs and NRTs published up to March 1, 2024.A comprehensive search strategy (Supplemental Digital 1) was developed for this systematic review, utilizing a combination of Medical Subject Headings (MeSH) and text words for each database. In addition to database searching, as a supplementary step, we conducted a manual exploration of the references within the selected articles.

### Data extraction

Two reviewers, Yaqiong Wang and Zha Peng, independently extracted and assessed the following data: author’s first name, year of publication, study design, patient characteristics, interventions, and outcomes. Any disagreements were resolved through discussion and consensus, with a third investigator, Zhenkun Tan, serving as an arbiter if necessary.

### Quality assessment

A modified Newcastle-Ottawa Scale (NOS) (15) was employed to evaluate the methodological quality of the non-randomized studies. Studies receiving >7 stars were categorized as high quality, 4–6 stars as medium quality, and <4 stars as low quality. We used Cochrane risk-of-bias tool for randomized trials version 2 for quality assessment of RCTs.

### Statistical analysis

Meta-analysis was performed using Review Manager 5.4 (The Nordic Cochrane Centre, Copenhagen, Denmark). Dichotomous data was evaluated using odds ratios (ORs) and 95% confidence intervals (CIs), while standardized mean differences were used for continuous outcomes such as hospital stay, when data were presented as medians and ranges instead of means and standard deviations (SD) for continuous variables, we converted these values to means and SD using the formula described by Luo et al (16). Heterogeneity was assessed using the chi-squared (χ²) and I² statistics. Initially, a fixed-effects model was considered if heterogeneity was not statistically significant (I² ≤ 50%, P >.10). However, the final decision on the model was also based on the clinical and methodological characteristics of the included studies. If significant clinical or methodological heterogeneity was identified, a random-effects model was employed to provide a more conservative estimate of the effect size, even if the I² value was below the threshold. Sensitivity analysis was conducted to assess the influence of individual studies with high heterogeneity (I² ≥ 75%) on the pooled estimates. Statistical significance was defined as P <.05. Publication bias was not assessed because <10 studies were included in the meta-analysis.

## Results

### Literature screening process

**Figure 1:**
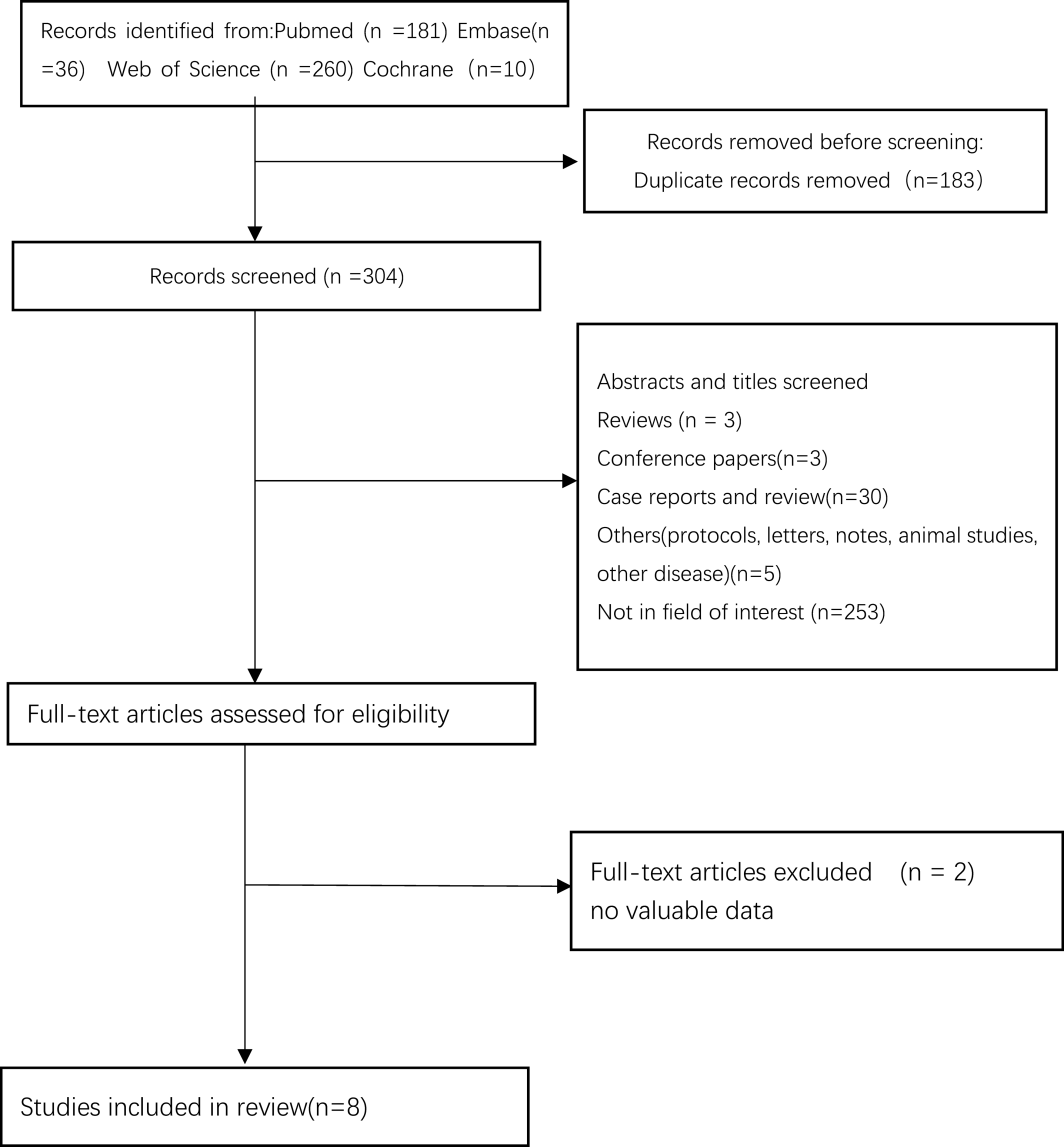
Flow chart of selection process.

**Figure 1** shows the literature screening process. A total of 487 studies were retrieved from the initial literature search. After eliminating duplicate records and conducting a title and abstract screening, 10 studies were deemed eligible for full-text review. Subsequent full-text assessment resulted in the inclusion of 8 studies (10, 11, 17–22). A total of 1059 patients (PRFA 603, LRFA 456) were included in the meta-analysis. The characteristics of the included studies are summarized in **Table 1**. All of studies exhibited a NOS score exceeding 7. (**Table 2**). Geographically, the studies were distributed across East Asia, with four conducted in China, three in Korea, and one in Japan. Comparative analysis revealed no statistically significant differences between the two groups with respect to age, gender, laboratory findings (specifically serum AFP and PIVKA-II levels), Child–Pugh classification, and tumor diameter.

**Table 1:** The baseline characteristics of the included studies.

Studies	Year	Research type	Study area	Number of patients		Gender(male/female)		Age(year)		Tumor diameter(cm)		HBV carrier		Liver cirrhosis		Child-Pugh grade(A/B)	
				LRFA	PRFA	LRFA	PRFA	LRFA	PRFA	LRFA	PRFA	LRFA	PRFA	LRFA	PRFA	LRFA	PRFA
Hong Jae Jeon. et al. (20)	2023	Retrospective study	Korea	151	156	116/35	100/56	63.60	62.35	1.73	1.59	83	68	135	116	138/13	146/10
Hyuk Soo Eun. et al. (10)	2017	Retrospective study	Korea	71	173	48/23	111/62	63.0	62.5	1.8	1.7	38	79	60	129	50/20	147/25
Wei Dai. et al.	2023	Retrospective cohort study	China	69	40	56/13	36/4	NR	NR	NR	NR	NR	NR	40	23	55/14	38/2
Huaiyin Ding. et al. (17)	2017	Retrospective study	China	56	60	40/16	38/22	55.2 ± 9.5	53.7 ± 6.8	2.31± 0.37	2.24 ± 0.33	46	50	35	41	34/22	35/25
Masashi Hirooka.et al. (18)	2009	Retrospective study	Japan	37	37	20/17	27/10	66.6 ± 7.9	70.2 ± 8.5	2.60 ± 0.69	2.49 ± 0.46	6	6	NR	NR	25/12	29/8
Min Hwan Kwak. et al. (19)	2022	Retrospective study	Korea	23	30	18/5	21/9	61.2 ± 8.0	56.7 ± 11.3	1.6 (1.3–1.9) #	1.9 (1.6–2.1) #	17	23	NR	NR	17/1	23/4
Wei Zhang. et al. (21)	2016	Retrospective study	China	19	77	17/2	64/13	55.3 ± 10.7	53.5 ± 12.8	2.30 ± 0.6	2.30 ± 0.6	15	64	NR	NR	18/NR	74/NR
CHEN Shude. et al. (22)	2018	Retrospective study	China	30	30	NR	NR	NR	NR	NR	NR	NR	NR	NR	NR	NR	NR

#, median (range); NR, not reported; HBV, Hepatitis B virus.

**Table 2:** The Newcastle-Ottawa Scale (NOS) score of the literature.

Studies	Selection				Comparability	Exposure			NOS score
	Is the case definition adequate	Representativeness of the cases	Selection of Controls	Definition of Controls	Comparability of cases and controls on the basis of the design or analysis	Ascertainment of exposure	Same method of ascertainment for cases and controls	Non-Response Rate	
Hong Jae Jeon (20)	1	1	0	1	2	1	1	1	8
Hyuk Soo Eun (10)	1	1	0	1	2	1	1	1	8
Huaiyin Ding (17)	1	1	0	1	1	1	1	1	7
Wei Dai (11)	1	1	0	1	1	1	1	1	7
Masashi Hirooka (18)	1	1	0	1	1	1	1	1	7
Min Hwan Kwak (19)	1	1	0	1	1	1	1	1	7
Wei Zhang (21)	1	1	0	1	1	1	1	1	7
CHEN Shude (22)	1	1	0	1	1	1	1	1	7

### Overall survival rates

**Figure 2:**
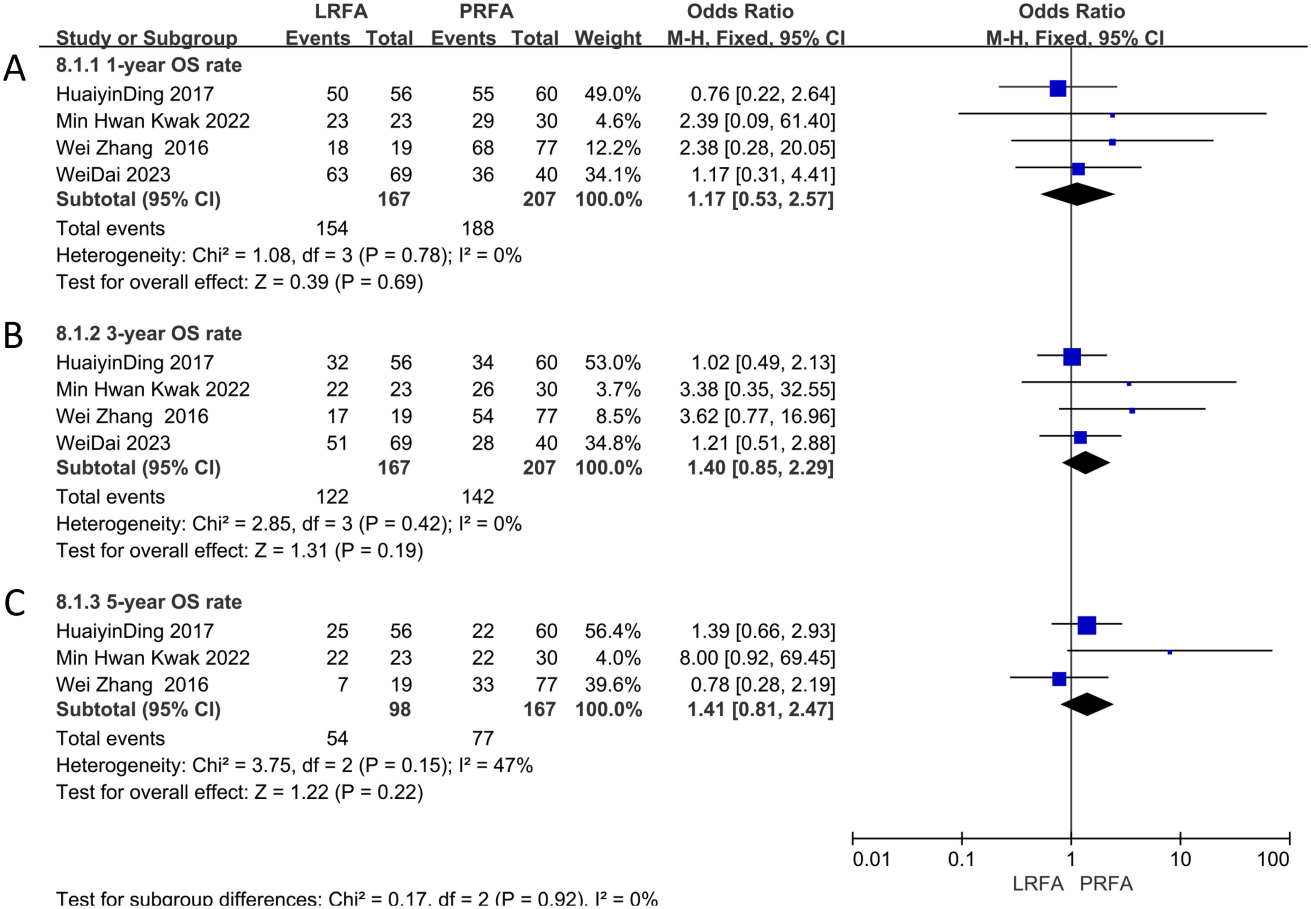
Forest plots of Overall survival (OS) between LRFA and PRFA on **(A)** 1-, **(B)** 3-, and **(C)** 5-y.

A total of four studies (11, 17, 19, 21) examined the 1-year OS data, and the meta-analysis demonstrated no significant difference between the two groups (OR: 1.17, 95% CI: 0.53-2.57, p = 0.69) (**Figure 2A**). The same studies (11, 17, 19, 21) also assessed 3-year OS rate, and the results indicated no significant difference (OR: 1.40, 95% CI: 0.85-2.29, p = 0.19) (**Figure 2B**). Additionally, three studies (17, 19, 21) evaluated 5-year OS data, and the meta-analysis showed no significant difference in the 5-year OS rate between the two groups (OR: 1.41, 95% CI: 0.81-2.47, p = 0.22) (**Figure 2C**). The heterogeneity analysis for the 1- and 3-year OS rates revealed no significant results (p = 0.78, I^2^ = 0%; p = 0.42, I^2^ = 0%, respectively), but moderate heterogeneity was observed in the 5-year OS rates (p = 0.15, I^2^ = 47%). The fixed effects model was used.

### Disease-free survival rates

**Figure 3:**
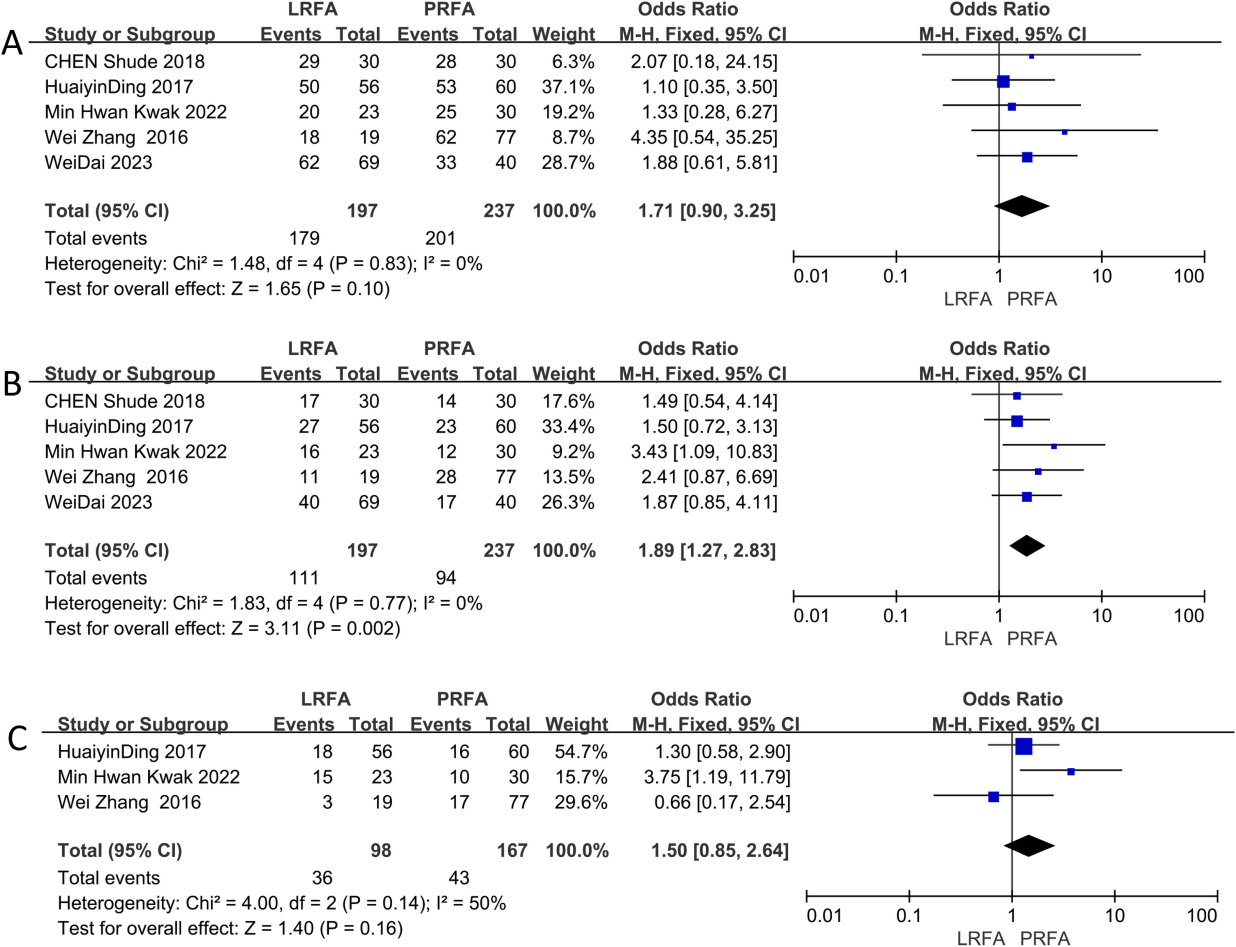
Forest plots of Disease Free Survival (DFS) between LRFA and PRFA on **(A)** 1-, **(B)** 3-, and **(C)** 5-y.

The 1-year DFS data were evaluated in five studies (11, 17, 19, 21, 22), and the results showed no significant difference between the LRFA and PRFA groups (OR: 1.71, 95% CI: 0.90-3.25, p = 0.10) (**Figure 3A**). However, the meta-analysis of 3-year DFS rates from 5 studies (11, 17, 19, 21, 22) revealed a significantly higher rate in the LRFA group (OR: 1.89, 95% CI: 1.27-2.83, p = 0.002) (**Figure 3B**). Additionally, the 5-year DFS rates from three studies (17, 19, 21) showed no significant difference between the two groups (OR: 1.50, 95% CI: 0.85-2.64, p = 0.16) (**Figure 3C**). The analysis of heterogeneity revealed no significant differences in the 1- and 3-year DFS rates (p = 0.83, I^2^ = 0%; p = 0.77, I^2^ = 0%, respectively), but moderate heterogeneity was observed in the 5-year DFS rates (p = 0.14, I^2^ = 50%). The fixed effects model was used for the analysis.

### Length of hospital stay

**Figure 4:**
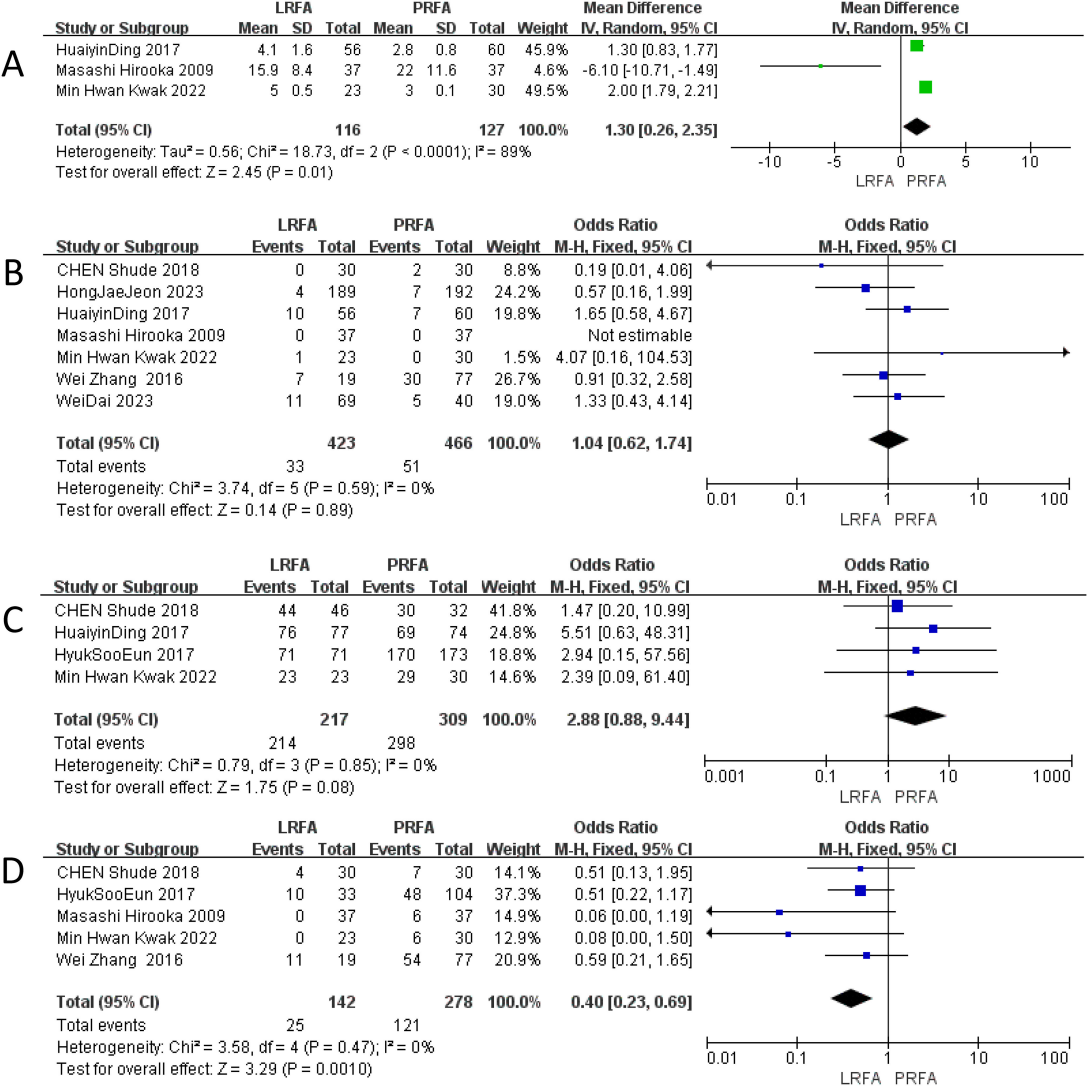
Forest plots of LRFA vs. PRFA on **(A)** length of hospital stay, **(B)** overall complication rates, **(C)** ablation success rates, **(D)** local recurrence rate.

Three studies reported data regarding the length of hospitalization (17–19). PRFA was associated with a significant reduction in the length of hospital stay (MD = 1.30; 95% CI 0.26 to 2.35; p=0. 01) (**Figure 4A**). Heterogeneity was significant (I^2^ = 89%; p<0.0001), the random-effects model was used. After excluding the any single study, and heterogeneity still exists.

### Overall complication rates

A total of seven studies (11, 17–22) with 889 patients (423 in the LRFA group and 466 in the PRFA group) provided data on overall complications. The results of the meta-analysis showed no significant difference in the overall complication rate between the two groups (OR: 1.04, 95% CI: 0.62 to 1.74, p = 0.89). The analysis revealed no significant heterogeneity among the studies, the fixed effects model was used (**Figure 4B**).

### Ablation success rates

Four studies (17, 19, 20, 22) provided data on the ablation success rate. The reported ablation success rate was higher for the laparoscopic ablation (LRFA) group compared to the percutaneous ablation (PRFA) group in these 4 studies. The meta-analysis showed that the odds of a successful ablation were higher for the laparoscopic procedures compared to the percutaneous procedures, although this difference did not reach statistical significance (OR 2.88, 95% CI 0.88–9.44, p = 0.08). No significant heterogeneity was observed in this analysis, the fixed effects model was used. (**Figure 4C**).

### Local recurrence rates

The local recurrence rate was evaluated in five studies (10, 18, 19, 21, 22), and the results indicated a significantly lower rate in the LRFA group compared to the PRFA group (OR: 0.40, 95% CI: 0.23-0.69, p = 0.0010). No significant heterogeneity was observed among the included studies, suggesting consistent findings across the studies, the fixed effects model was used (**Figure 4D**).

## Discussion

Ablation techniques, particularly radiofrequency ablation (RFA) and microwave ablation (MWA), have emerged as widely used minimally invasive approaches. Over the past decade, several meta-analyses have been conducted in the field of ablation therapies to determine the most effective and safe treatment options for HCC. In comparison to the previous meta-analyses conducted by Joslin R. Musick in 2023 (23), Qian Yu in 2021 (24), Moustafa Abdalla in 2023 (25), and Tan in 2019 (26), our study focuses specifically on comparing LRFA and PRFA for HCC, excluding the influence of different ablation methods on the outcomes.

In our meta-analysis, LRFA showed significantly higher 3-year DFS rates and a lower local recurrence rate. Additionally, PRFA was associated with slightly shorter postoperative hospital stays compared to LRFA. However, no significant differences were observed between LRFA and PRFA in terms of OS rates at 1, 3, and 5 years, overall complication rates, ablation success rates, and 1- and 5-year DFS rates, indicating that both techniques are safe and effective for treating HCC.

Several factors may contribute to the superior 3-year DFS rates and lower local recurrence rate observed with LRFA compared to PRFA. Firstly, LRFA provides a direct visual inspection of the liver under laparoscopic guidance, allowing for a more comprehensive surgical view. Second, LOUS can help identify satellite lesions or additional tumors that may not have been detected on pre-procedure imaging (27). Santambrogio et al. (28) further underscore LOUS during LRFA identified 25% of HCC lesions missed by preoperative imaging. By detecting these tumors during the procedure, the physician can adjust the treatment plan to ensure that all tumor tissue is adequately treated, which can improve treatment outcomes and reduce the risk of recurrence. Third, LRFA enables the Pringle maneuver, reducing hepatic blood flow and expanding the injury area through thermal coagulation. This allows for the ablation of tumors near major blood vessels with minimal hemorrhage risk (29). During the procedure, CO2 infusion increases intraabdominal pressure, significantly decreasing intrahepatic blood flow due to vascular collapse, which enhances ablation effectiveness (30). Fourth, LRFA can reduce the impact of rib obstruction on puncture angles, allowing for the selection of more optimal puncture angles to effectively cover liver cancer lesions, especially for tumors located in special positions (31).

Although LRFA demonstrated better 3-year DFS rates, no significant differences in OS rates and 1-year and 5-year DFS rates were observed between LRFA and PRFA. Specifically, in the analysis of 5-year DFS rates, significant heterogeneity was observed among the included studies. Given the limited number of studies (only three) in this analysis, subgroup analyses could not be conducted. However, sensitivity analysis identified the study by Min Hwan Kwak (19) as the primary source of heterogeneity. The extended follow-up period in this study may have provided a more accurate assessment of DFS but could also have introduced temporal bias. This highlights the importance of considering the duration of follow-up when interpreting DFS outcomes.

The significant reduction in hospital stay for PRFA is an important clinical advantage. This finding can be attributed to several factors. Firstly, PRFA is a less invasive procedure that does not require general anesthesia, which reduces the need for postoperative monitoring and recovery time. Secondly, the absence of surgical incisions and the lower risk of intra-abdominal complications associated with PRFA contribute to shorter hospital stays. This not only reduces the burden on healthcare resources but also decreases the economic burden on patients and their families. Additionally, shorter hospital stays can improve patient satisfaction and quality of life, particularly for elderly patients or those with multiple comorbidities. we observed heterogeneity in the analysis of hospital stay duration. Even after excluding any single study, the heterogeneity persisted, indicating that multiple factors may be contributing to this variability. Given the limited number of studies, subgroup analysis was not feasible. The differences in hospital stay duration may be related to the treatment modality, postoperative management, and the criteria for discharge used by different medical institutions.

The quality of the studies included in meta-analysis is a critical factor that may contribute to heterogeneity. Despite similarities in baseline patient characteristics across the studies, uncontrolled variables may influence the outcomes. Additionally, differences in specific interventions and measurement methods used in each study could lead to variability in the results. Therefore, we urge caution in interpreting these findings.

LRFA offers distinct technical advantages. Recent advancements in LOUS have further enhanced the accuracy of intraoperative tumor detection. The combined use of LRFA with laparoscopic liver resection for patients with multiple lesions, where larger lesions are resected and smaller lesions are ablated, represents a promising integrated approach increasingly recognized in the management of HCC. PRFA also offers several advantages. Firstly, its lower invasiveness results from the absence of incisions or laparoscopic access, leading to minimal trauma. This is particularly beneficial for patients who cannot tolerate surgery or are at risk of intra-abdominal adhesions. Secondly, PRFA is associated with a significantly shorter hospital stay, which not only reduces the utilization of medical resources but also provides economic benefits for patients. Additionally, PRFA can typically be performed under local anesthesia or sedation, thereby minimizing the risks associated with general anesthesia, such as cardiopulmonary complications. This is especially advantageous for elderly patients with multiple comorbidities. Lastly, while direct cost comparisons are lacking in current studies, PRFA’s shorter hospital stay and less complex procedural requirements suggest potential cost-effectiveness.

Our study has several limitations. First, the small sample size may indeed limit the statistical power. Additionally, not all studies reported critical outcomes. This reflects the inherent limitations of the original studies, where retrospective designs often prioritize specific outcomes based on clinical focus or data availability. The variability in reported outcomes stems from differences in study designs and objectives. Furthermore, heterogeneity across outcomes was evident, and there were indications of publication bias in multiple studies. It is crucial to undertake larger, long-term comparative studies and prioritize well-designed randomized controlled trials to validate the current findings. This will provide more robust evidence for clinical decision-making.

In summary, both LRFA and PRFA are viable treatment options for HCC, each with its own set of advantages. LRFA appears to be more effective in terms of oncologic outcomes, particularly in reducing local recurrence and improving mid-term DFS. The advantages of PRFA, including its lower invasiveness, shorter hospital stay, avoidance of general anesthesia, and potential cost-effectiveness. The choice between LRFA and PRFA should be individualized based on tumor characteristics, patient comorbidities, and the availability of technical expertise. Future research should focus on large-scale, prospective, multicenter trials to further validate these findings and explore the optimal integration of these techniques within a comprehensive liver cancer management strategy.

## Data Availability

The original contributions presented in the study are included in the article/**Supplementary Material**. Further inquiries can be directed to the corresponding author.
