# Supplementary material for: A systematic review and meta-analysis of the comparison of laparoscopic radiofrequency ablation to percutaneous radiofrequency ablation for hepatocellular carcinoma
Source: Front Oncol. 2025 Mar 11;15:1559343. doi: 10.3389/fonc.2025.1559343 (PMC11932898; doi:10.3389/fonc.2025.1559343)
Supplement: Supplementary file 1 [file DataSheet1.docx]

Searching strategy

1.(((("Carcinoma, Hepatocellular"[Mesh] OR (hepatocellular[All Fields] AND carcionoma[Text word])) OR hepatocarcinoma[Text word]) OR liver cell carcinoma[Text word]) OR liver cell cancer[Text word]) OR primary liver cell cancer[Text word]

**2 laparoscopic RFA OR L-RFA OR LRFA laparoscopy-assisted radiofrequency ablation**

**Laparoscopic radiofrequency ablation**

**3 percutaneous radiofrequency ablation percutaneous RFA PRFA**

**Pubmed**

1.(((("Carcinoma, Hepatocellular"[Mesh] OR (hepatocellular[All Fields] AND carcionoma[Text word])) OR hepatocarcinoma[Text word]) OR liver cell carcinoma[Text word]) OR liver cell cancer[Text word]) OR primary liver cell cancer[Text word] **(n=**115,101**)**

2. **((((laparoscopic RFA) OR (L-RFA)) OR (LRFA)) OR (laparoscopy-assisted radiofrequency ablation)) OR (Laparoscopic radiofrequency ablation)** **(n=1275)**

3. **((percutaneous radiofrequency ablation) OR (percutaneous RFA)) OR (PRFA) (n=6728)**

4. 1 and 2 and 3

**(((((((Carcinoma, Hepatocellular) OR (hepatocellular)) OR (hepatocarcinoma)) OR (liver cell carcinoma)) OR (liver cell cancer)) OR (primary liver cell cancer)) AND (((((Laparoscopic radiofrequency ablation) OR (laparoscopic RFA)) OR (L-RFA)) OR (LRFA)) OR (laparoscopy-assisted radiofrequency ablation))) AND (((percutaneous radiofrequency ablation) OR (percutaneous RFA)) OR (PRFA)) n=181**

**Embase**

1.**'liver cell carcinoma'**/exp **(n=228,007)**

2.**'liver cell carcinoma'**:ab,ti OR **'hepatocellular carcinoma'**:ab,ti OR **'liver cell cancer'**:ab,ti OR **hepatocarcinoma**:ab,ti OR **'primary liver cell cancer'**:ab,ti **(n=184,508)**

3. 1 OR 2 **(n=**251,553**)**

4. **'Laparoscopic radiofrequency ablation'**/exp **(n=37)**

5. **'Laparoscopic radiofrequency ablation '**:ab,ti OR **' laparoscopic RFA'**:ab,ti OR **' L-RFA '**:ab,ti OR **' LRFA '**:ab,ti **(n=417)**

6.4 OR 5 **(n=424)**

7. **'percutaneous radiofrequency ablation '**/exp **(n=129)**

8. **'percutaneous radiofrequency ablation'**:ab,ti OR **'percutaneous RFA'**:ab,ti OR **'PRFA'**:ab,ti **(n=2857)**

9. 7 OR 8 **(n=2877)**

10. 3 AND 6 AND 9 **(n=36)**

**Web of Science**

1.((((TS=(Carcinoma, Hepatocellular)) OR TS=(hepatocellular)) OR TS=(liver cell carcinoma)) OR TS=(liver cell cancer)) OR TS=(primary liver cell cancer) **(n=408,706)**

2.(((TS=(Laparoscopic radiofrequency ablation)) OR TS=(laparoscopic RFA)) OR TS=(L-RFA)) OR TS=(LRFA) **(n=1,831)**

3.((TS=(percutaneous radiofrequency ablation)) OR TS=(percutaneous RFA)) OR TS=(PRFA) **(n=10,536)**

4 .1 AND 2 AND 3 **(n=260)**

**Cochrane library**

**#1 (Carcinoma, Hepatocellular):ti,ab,kw OR ("hepatocellular carcinoma"):ti,ab,kw OR (hepatocarcinoma):ti,ab,kw OR (liver cell carcinoma):ti,ab,kw OR (liver cell cancer):ti,ab,kw(n=10304)**

**#2 - (laparoscopic RFA):ti,ab,kw OR (L-RFA):ti,ab,kw OR (LRFA):ti,ab,kw OR (laparoscopy-assisted radiofrequency ablation):ti,ab,kw OR (Laparoscopic radiofrequency ablation):ti,ab,kw(n=94)**

**#3 - (percutaneous radiofrequency ablation):ti,ab,kw OR (percutaneous RFA):ti,ab,kw OR (PRFA):ti,ab,kw(n=453)**

**#4 -**#1 and #2 and #3**(n=10)**
